# Case of Artificial Urethra and Puncture of the Bladder

**Published:** 1853-05

**Authors:** John D. Winston

**Affiliations:** Nashville


From the Southern Journal of the Medical and Physical Sciences.

*A Case of Artificial Urethra and Puncture of the Bladder.*—By John D. Winston, M. D., of Nashville.

Sometime in the Spring of 1815, and while living at Columbia, Ky., I saw R M. W., a youth aged about twenty years, the patient of my relation and partner, Dr. Caldwell, now of Louisville. He was suffering intensely from suppression of water, caused by stricture of the urethra, the result, as I understood from the doctor, of long continued and badly cured gonorrhœa. Finding the introdution of the catheter impracticable, he was soon relieved by means of the lancet, warm bath and nausients. The recurrence of the paroxysms had been frequent and severe, for which almost every variety of treatment had been used, such as bougies, cauteries, constitutional remedies, &c., Also the suggestions of Profs. Gross and Dudley, under whose care he had occasionally been, had all been brought to bear on his case, without the least prospect of anything like permanent relief. Under these circumstances, Dr. C. proposed relief by a division of the strictures. Accordingly, in a few days, Dr. Harding and myself being present, he operated, dividing two strictures, as well as I recollect, near the bulb of the urethra and attempted to introduce a catheter through which the urine, should pass while the parts might heal. But this, alas! he failed to accomplish; and as the patient was now urinating without difficulty, nothing further was attempted. Truly an unenviable condition, far worse than before the operation.

The second day after, and just forty-eight hours from this operation, during which time Dr. C. had not left him, I was hastily summoned to the case, found no water had passed for thirty-six hours, the bladder distended to its full extent, and his suffering, almost intolerable. To puncture the bladder through the rectum, at which point it could easily be felt, or by the upper operation, would have afforded at best but momentary relief. In this emergency we proposed forcible catheterism, as described by Brodie, a division of the parts as near in the direction of the natural passage as practicable, back and into the bladder.

At the request of Dr. C. I operated, by placing my left forefinger in the rectum, the other on the top of a spear pointed bistoury, dividing the parts back till the tense tympanitic bladder was felt, which I entered with a bilateral sweep, dividing, as I supposed, the seminal vessels, which time has proven was done. The water discharged, a catheter was introduced from the end of the penis through the new opening into the bladder, and there retained forty days, the time required for the complete healing and continuity of the parts. After the operation I saw but little of the case; leaving it almost exclusively in the care of Dr. C, by whose skill and unremitting attention he was soon recovered, has ever since been free of any thing like stricture, has married, is still living, and the father of several children.

I took no notes of this case at the time it occured; and although to my mind it presents some points of interest, should not now, or perhaps ever have published it, but for the fact of a casual conversation I had a few days since with a much esteemed and able practitioner of this city, in reference to a case then under his treatment; the management of which involved a point he doubted the practicability of making, and as I conceive was fully settled in the cure of my patient. The case to which I alluded was one of imperforate anus, in which he feared that if an operation was made so as to reach the bowel, it would remain an irritating and suppurating surface, unprotected by mucus membrane, and an involuntary flow of the fœces for want of a proper sphincter, would be the result.

I enterta n no such doubts ; nothing of the kind certainly ever occured in the above case. For the newly formed portion of the urethra, although made through ordinary muscle, cellular tissue, &c., healed rapidly, and was when healed and has continued to be as well protected by mucus membrane as any other portion of the urethra ; otherwise it would, at some time, have exhibited some signs of irritation, which, so far as I am informed, has never been the case. Nor has incontinence of the urine ever occurred, proving conclusively, that although the bladder, in its highly distended state, with the neck thrown far upwards and backwards, must have been entered considerably below the neck, perhaps not less than from one to two inches, yet at that point a sphincter was formed, or something that answered the purpose as well. Even more. The divided vassa defferens, accomodated themselves to the new opening, performing their functions in common with every other part concerned, as harmoniously and healthily as though nothing had occurred. Otherwise emasculation in effect would have been the result, and he could not have propagated his species as he has done.

